# The role of microbiota in nonalcoholic fatty liver disease: mechanism of action and treatment strategy

**DOI:** 10.3389/fmicb.2025.1621583

**Published:** 2025-07-30

**Authors:** Siwen Shen, Yao Liu, Nuoya Wang, Zhenhe Huang, Guifang Deng

**Affiliations:** ^1^Department of Clinical Nutrition, Shenzhen Nanshan People’s Hospital, Shenzhen, China; ^2^Department of Geriatric Medicine, Shenzhen Nanshan People’s Hospital, Shenzhen, China

**Keywords:** NAFLD (non-alcoholic fatty liver disease), metabolic associated fatty liver disease (MAFLD), prebiotics and probiotics, gut-liver axis, bile acid

## Abstract

Non-alcoholic fatty liver disease (NAFLD) is now the most prevalent chronic liver disease worldwide, ranging from simple hepatic steatosis to non-alcoholic steatohepatitis (NASH) and hepatocellular carcinoma. It poses a significant public health challenge. Growing evidence indicates that the gut microbiota plays a key role in the development and progression of NAFLD. Advances in sequencing technologies, microbiome and metabolomics have helped identify characteristic microbial patterns and microbial-derived metabolites associated with NAFLD. The gut-liver axis has emerged as a central pathway linking intestinal microbes to liver function. Microbiota-derived metabolites, such as short-chain fatty acids, bile acids (BAs), and trimethylamine N-oxide (TMAO), have dual roles in hepatic lipid accumulation, inflammation, and insulin resistance, providing new insight into NAFLD pathogenesis. This review summarizes the mechanisms by which disruptions in the gut-liver axis contribute to NAFLD progression. It also outlines the therapeutic effects and mechanisms of current probiotics, with particular emphasis on next-generation probiotics like *Akkermansia muciniphila* and the potential benefits of its inactivated forms. Furthermore, we explore the role of prebiotics, plant-derived compounds, and synthetic agents in modulating gut microbiota and liver health. The review highlights key associations between specific bacterial species, microbial metabolites, and NAFLD, offering a theoretical basis for microbiota-targeted precision interventions and new therapeutic directions.

## Introduction

### NAFLD

Non-alcoholic fatty liver disease (NAFLD) is a spectrum of liver condition characterized by macrovesicular accumulation of triglyceride in hepatocytes without the evidence for ongoing or recent consumption of significant amounts of alcohol (Chalasani et al., 2018). The redefinition of NAFLD as metabolic associated fatty liver disease (MAFLD) reflects its strong association with metabolic disorders, including obesity, dyslipidemia, insulin resistance, hypothyroidism, and obstructive sleep apnea (Eslam et al., 2020). NAFLD is recognized as the most prevalent liver disorder, with a global prevalence of 25% (Younossi et al., 2016). Several systematic reviews have demonstrated that NAFLD affects over 33% of the Asian population as of 2017 with an increasing incidence rate of 29.7 per 1,000 person-years. The progression of NAFLD follows a well-defined spectrum from benign simple steatosis, non-alcoholic fatty liver (NAFL), steatohepatitis and to more advanced disease called non-alcoholic steatohepatitis (NASH) with advanced fibrosis and cirrhosis, ultimately lead to hepatocellular carcinoma, liver failure, and death. The presence of NASH can increase risk to develop fibrosis, cirrhosis or hepatocellular carcinoma. American Association for the Study of Liver Diseases defines NAFL as the presence of greater than 5% hepatic steatosis without evidence of hepatocellular injury while NASH is defined as the presence of >5% hepatic steatosis with inflammation and hepatocyte injury (Chalasani et al., 2018). So far, the pathogenetic mechanisms of NAFLD are complex and not yet fully elucidated.

Noticeably, only a few guidelines recommended pharmacological treatment are available. For example, PPAR-γ ligand drug Pioglitazone, which is primary focus on blood glucose control for diabetes, helps control liver damage (Chalasani et al., 2018; Sheka et al., 2020). Several new medications are currently under research targeting on NAFLD, particularly for its more severe form, NASH. Rezdiffra (resmetirom), a newly FDA-approved treatment for NASH, functions as a thyroid hormone receptor beta (THR-β) agonist, which is thought to aid in reducing liver fat and improving fibrosis in NASH patients (Harrison et al., 2024). Nutritional supplements such as vitamin E at a dose of 800 IU daily and omega-3 fatty acid at a dose of 1 g daily have demonstrated positive effects in reducing inflammation, decreasing fat accumulation and improving liver histology (Sanyal et al., 2010; Nogueira et al., 2016). Apart from medications, the treatment of NAFLD is highly rely on lifestyle intervention at the early stage, with the main objective being weight loss and healthy weight maintenance. Several studies has been demonstrated that a 5% weight reduction is required to decrease hepatic steatosis, while weight loss of 7%–10% can help to improve liver inflammation and fibrosis, even complete resolution of their NASH (Vilar-Gomez et al., 2015). Current pharmacological management of NAFLD faces limited therapeutic options and inability to comprehensively address its multifactorial pathogenesis. Notably, accumulating evidence has demonstrated the therapeutic efficacy of gut microbiota modulation in obesity and type 2 diabetes mellitus (T2DM), providing novel insights for NAFLD intervention (Ng et al., 2022; Zhang et al., 2025).

Currently, it is believed that NAFLD tends to be a multifactorial disease. It involves genetic, metabolic, and environmental factors, including epigenetic modifications, dietary intake, hormones secreted by adipose tissue (leptin, adiponectin), crosstalk or organization between different organs, etc (Trépo and Valenti, 2020). Genome-wide association studies (GWAS) have identified several genetic variants, such as *PNPLA3, TM6SF2, MBOAT7* are strongly associated with the severity of NAFLD (Longo et al., 2021). Epigenetic modifications, such as DNA methylation and miRNA regulation, lead to abnormal expression of key metabolic genes, while dysregulation of miRNAs can impact lipid metabolism and inflammatory responses (Juanola et al., 2021). For example, hypomethylation of fibrogenic genes such as TGF-β1, Collagen 1A1 and platelet-derived growth factor, has been observed in advanced stages of NAFLD, accompanied by their transcriptional upregulation to exacerbate lipid accumulation and fibrosis. Moreover, the gut microbiota can recruit DNA methyltransferase 3 (DNMT3) to induce epigenetic modifications of Toll-like receptor 4 (TLR4) in intestinal epithelial cells, thereby impacting hepatic fibrosis and steatosis (Tang et al., 2024). In addition, microbial metabolites TMAO upregulate members of the miR-17/92 cluster in liver, which in turn enhances the expression of target genes associated with inflammation, thus promoting NAFLD progression.

Environmental factors, including high-calorie diets, sedentary lifestyles, smoking, and air pollution, exacerbate NAFLD progression by inducing oxidative stress and inflammation. These factors interact with each other, collectively driving the evolution of NAFLD from simple steatosis NASH and even liver fibrosis (Alferink et al., 2019; Geier et al., 2021; Juanola et al., 2021). Among these risk factors, a growing body of evidence indicates that gut–liver axis is implicated in the onset and progression of NAFLD (Knudsen et al., 2019; Michels et al., 2022). Given the microbiota’s plasticity, targeting gut microbiota composition presents a synergistic effects between microbial interventions and conventional therapies to develop promising therapeutic approach for NAFLD.

### Gut microbiome

In recent years, groundbreaking research has illuminated the profound connection between gut microbiota and metabolic diseases, revealing a complex interplay that extends far beyond traditional understanding. Comprehensive investigations have established that the gut microbiome is integral to metabolic regulation, affecting various conditions including obesity, T2DM, and NAFLD (Dugas et al., 2018).

The process of human gut microbiomes colonization starts prenatally and develop across the intestine in early life, therefor to shape physiological and immunological functions throughout an individual’s life (Rodríguez et al., 2015). Approximately 150 to 400 species reside in each person’s gut with mostly species belong to the Bacteroidetes, Firmicutes, Actinobacteria, and Proteobacteria phyla (Lloyd-Price et al., 2016; Davenport et al., 2017). The composition of gut microbiota is continuously adaptive and dynamic with unhealthy dietary habits, sedentary lifestyle, antibiotics usage, and toxic chemicals exposure can promote sustained modifications of the gut ecosystem homeostasis, leading to dysbiosis. Accumulating evidence demonstrates significantly reduced overall bacterial diversity and richness in NAFLD patients compared to healthy controls. Critically, diminished α-diversity and restructured β-diversity are consistently observed in NAFLD cohorts, with both metrics correlating with disease severity progression. (Shen et al., 2017; Tsai et al., 2020; Zeng et al., 2024). Dysbiosis has been linked to NAFLD through various mechanisms. It is believed that the microbiota profile can influence intestinal permeability, allowing bacteria or their derived factors to reach the liver and contribute to liver injury, steatohepatitis, and fibrosis progression (de Vos et al., 2022; Michels et al., 2022; Hsu and Schnabl, 2023).

A large number of studies has been focusing on the composition of the gut microbiota in fecal samples among NAFLD patients and related metabolic disease condition. Among animals and human studies, the abundance of certain strains such as *Lactobacillaceae, Christensenellaceae*, and *Intestinibacter* have been found to be negatively correlated with NAFLD, while others like *Coriobacteriia, Actinomycetales*, and *Oxalobacteraceae* show positive associations with the disease (Li Y. et al., 2023). Moreover, the abundance of *Bacteroides*, especially *B. uniformis and B. bifidum* were reported markedly decreased in the NAFLD group (Demir et al., 2020; Xu et al., 2024). At phylum level, one German cross-sectional prospective study found higher abundances of Gram-positive Actinobacteria and Firmicutes comparing with healthy controls. However, the shift of gut microbiota was not consistent among studies. A higher level of the Bacteroidetes and lower levels of Firmicutes and Lentisphaerae were detected among NAFLD patients than in controls in Asian population (Tsai et al., 2020). While the Firmicutes/Bacteroidetes ratio had been reported as a biomarker for obesity, it does not significantly correlate with fibrosis or steatosis in groups other than the obese group (Magne et al., 2020; Jasirwan et al., 2021).

Fewer studies have focused on the changes in gut microbiome profiles during the progression from NAFLD to NASH as well as the advanced degree of fibrosis as determined by liver histology. A decreased in Firmicutes and *Faecalibacterium prausnitzii*, along with an increase of Proteobacteria and *E. coli* abundance has been observed in patients with advanced NASH fibrosis (Loomba et al., 2017). Additionally, a recent study also indicated hepatic steatosis was associated with lower microbial alpha diversity and the presence of *Coprococcus* and *Ruminococcus gnavus* (Alferink et al., 2021). The association between gut microbiota and NAFLD is summarized in Table 1.

**TABLE 1:** Association between gut microbiota and NAFLD.

Category	Name	Abundance association with NAFLD	Key observations	Proposed mechanisms	References
Phylum	Actinobacteria	Positive	Increase in German	Increase lipid accumulation	Tsai et al., 2020
			NAFLD cohort	Dysregulated bile acid metabolism	
	Bacteroidetes	Controversial	Increase in Asian NAFLD patients	Improving insulin resistance	Tsai et al., 2020; Yu et al., 2025
			Decrease in Western obesity study	Promoting tight-junction	
	Firmicutes	Controversial	Increase in German NAFLD cohort	Induce energy harvest	Magne et al., 2020
			Decrease in Asian NAFLD cohort	Decrease SCFA production	
	Lentisphaerae	Negative	Decrease in Asian NAFLD patients	Unknown metabolic functions	Tsai et al., 2020
	Proteobacteria	Positive	Increase in advanced	Enhanced endotoxin	Vasques-Monteiro et al., 2021
			NASH fibrosis	Decrease gut barrier integrity	
Genus/species	*Bacteroides uniformis*	Negative	Decrease in NAFLD cohorts	Increased CD107a to restore NK cell function	Xu et al., 2024
	*Bacteroides bifidum*			Increase hepatic NK cells	
	*Christensenellaceae*	Negative	Inversely correlated with hepatic fat content	Induce insulin resistance	Li Y. et al., 2023
				Induce fat accumulation	
	*Coprococcus*	Negative	Decrease in NASH fibrosis	Enhance hepatocyte ballooning	Alferink et al., 2021
				Induce inflammatory profile	
	*Coriobacteriia*	Positive	Increase in NAFLD patients	Dysregulate lipid metabolism	Mu et al., 2020
	*Escherichia/Shigella*	Positive	Increase in advanced NASH fibrosis	Induce endotoxemia; increase ethanol concentrations	Frost et al., 2021
				Increased intestinal permeability	
	*Faecalibacterium prausnitzii*	Negative	Decrease in advanced NASH fibrosis	Reduced SCFAs production	Shin et al., 2023
				Dysregulated glucose metabolism	
	*Intestinibacter*	Negative	Decrease in NAFLD patients	Exacerbate insulin resistance	Li Y. et al., 2023
	*Lactobacillaceae*	Negative	Decrease in NAFLD patients	Suppress SCFAs production	Li Y. et al., 2023
	*Ruminococcus gnavus*	Positive	Increase in hepatic steatosis patients	Reduce gut microbial diversity	Alferink et al., 2021
				Reduce the production of acetate	

However, it still remains uncertain whether the altered gut dysbiosis observed in NAFLD is merely a consequence results of the disease or if it actually contributes to the disease process itself. A longitudinal study conducted in Germany demonstrated that long-term instability of the gut microbiome over a 5-year interval with dominance of *Enterobacteriaceae* and *Escherichia/Shigella* correlated with development of NAFLD and T2DM, suggesting that participants who later developed fatty liver disease or diabetes already showed significant microbiota changes at the outset, even before the diseases were clinically apparent (Frost et al., 2021).

Gut microbiota-derived metabolites critically regulate metabolic homeostasis. For instance, short chain fatty acids (SCFAs), which are primarily produced through the fermentation of dietary fibers by gut microbiota, play a crucial role in maintaining intestinal barrier integrity for gut-liver axis homeostasis. Once production in the gut, SCFAs enter the portal circulation, providing a direct communication channel between the intestine and liver. This allow SCFA to exert metabolic effect including lipid homeostasis and energy expenditure, as well as to activate AMPK pathways, regulating gluconeogenesis and improve insulin sensitivity (Hernández et al., 2019). Besides SCFAs, bile acids (BAs) which are primary produced in liver, play as signaling molecules to activate nuclear receptors, which can exacerbate lipid deposition and fibrosis. Along with imbalances in the gut microbiome that affect bile acid composition, contribute to the progression from simple fatty liver to a more advanced disease state (Perino et al., 2021). Furthermore, the gut microbiota’s role in NAFLD is also seen in its interaction with inflammation factors. Gut-derived pro-inflammatory metabolites can activate inflammatory cytokine signal pathways in the liver, leading to severe inflammation, fibrosis, and liver damage in NAFLD. This interaction between the gut microbiota and the host’s immune system is also a key factor in the pathogenesis of NAFLD (Hammerich and Tacke, 2023).

A growing body of research has revealed that supplementation with probiotics, such as *Bifidobacterium, Lactobacillus* and *Akkermansia*, are potential to restore the balance of gut microbiota and to improve the gut microecological environment (Mohamad Nor et al., 2021; Carpi et al., 2022). Additionally, probiotics can enhance gut barrier function, thereby alleviating liver inflammation and damage. For example, *Akkermansia muciniphila* modulate the expression of tight junction proteins and influence γδT cells and macrophages, which are two key hepatic innate immune cell populations (Han et al., 2023). There is clear strain-specificity for probiotic to become an important component in the treatment of NAFLD, and even emerge as one of the next-generation mainstream therapeutic approaches.

In this review, we provide a synopsis of the connection between the gut microbiota and NAFLD and highlight some recent proposed mechanisms in how gut microbes play a significant role in the development and progression of NAFLD, influencing the disease through its composition, metabolic activities, and potential future therapeutic directions. Understanding these relationships is crucial for developing potential therapeutic strategies focusing on microbiome modulation through probiotics, prebiotics, and personalized nutritional interventions, to prevent and treat NAFLD.

## Mechanism of intestinal microbiota affecting NASH and NAFLD

### Gut-liver axis

The gut-liver axis refers to the complex biochemical communication between the gut and liver cells, which plays a crucial role in maintaining health and in the progression of diseases. The portal vein delivers approximately 70% of the liver’s blood supply from the gut, which makes the liver the first line of defense against gut-derived substances. After digesting, nutrients and metabolites from the gut were transported to the liver to exert their physiological effects. As the largest mucosal surface of the human body, human gut is continuously exposed to dietary antigens and microbes (Hsu and Schnabl, 2023). This bidirectional relationship is mediated by several factors, including the gut microbiota composition, bile aids circulation, dietary components and circulated metabolites, cytokines etc. The liver influences the structure and function of the gut microbiota through bile secretion, while gut microbes and their metabolites affect hepatic metabolism and immune responses via the portal vein (Pabst et al., 2023). This dynamic between the gut and liver is central to the pathogenesis of NAFLD.

A healthy balanced microbiome is essential in terms of normal digestion and metabolic processes. However, in NAFLD, dysbiosis is commonly observed and can lead to the overgrowth of pathogenic bacteria and a decreased in beneficial microbiota, thus alter the production of metabolites that influence liver function. For example, dysbiosis result in the increased production of endotoxin lipopolysaccharide (LPS) (Di Ciaula et al., 2022). LPS later translocate from the gut to the liver through the portal circulation and activate Kupper cells, a specialized macrophages to regulate innate immune response, leading to inflammation and insulin resistance (Dixon et al., 2013; Wang G. et al., 2021). This phenomenon triggers a systemic inflammatory response, which is directed in the liver. Studies have shown that high-fat diets and excessive fructose intake can impair gut barrier function, increasing the risk of LPS entering blood stream, thus initiating pro-inflammatory cytokine release by activating Toll-like receptor 4 (TLR4) which in turn contribute to liver damage (Cho et al., 2021).

In the context of NAFLD, pro-inflammatory cytokines, including tumor Necrosis Factor (TNF), IFNγ, and IL-1β, and chemokines play key roles in regulating gut barrier function and immune responses. TNF-α promotes hepatocyte apoptosis, and activation of hepatic stellate cells (HSCs), which are involved in fibrosis development (Di Ciaula et al., 2022). Interleukin-1 beta (IL-1β) is another pro-inflammatory cytokine that contribute liver damage by inducing NF-kB pathway. IL-6 activates the JAK-STAT pathway, which leads to hepatocyte damage and fibrosis progression (Lokau et al., 2019). Given the interaction of cytokines within the gut-liver axis, they represent therapeutic targets. Modulating the levels of specific cytokines or their receptors could offer new strategies for treating NAFLD.

The gut-liver axis impacts metabolic function especially insulin resistance which is a hallmark in NAFLD individuals (Tilg et al., 2022). The gut microbiota influence insulin sensitivity by affecting bile acid metabolism, SCFA production, and by modulating the release of gut hormones like glucagon-like peptide 1 (GLP1) (Newsome et al., 2021). Dysbiosis also been shown to increase the production of secondary bile acid, that can disrupt liver function and promote lipid accumulation within hepatocytes (Di Ciaula et al., 2022). The gut-liver axis is involved in every stage of NAFLD from the onset of steatosis to the development of NASH, fibrosis and cirrhosis (Boulangé et al., 2016). Understand the mechanisms underlying gut-liver communication opens to new therapeutic avenues for treating NAFLD by targeting gut microbiota, gut barrier function, and inflammatory signaling pathways.

### Short chain fatty acid (SCFA)

Short chain fatty acids, mainly acetate, propionate and butyrate, are end-products of indigestible carbohydrates fermented by gut microbiome. They significantly influence the microbiota-host interaction within the gut-liver axis (Mann et al., 2024). While butyrate and propionate are extensively extracted and metabolized by the liver, a lesser percentage of acetate is taken up by the liver thus reaches the systemic circulation in significantly higher amounts to serve as a significant redistribute carbon source in humans (Bose et al., 2019; Moffett et al., 2020). Multiple factors can influence the production of SCFAs include dietary fiber intake, host health status, medication use, genetic factors, and lifestyle through modifying the microbiome composition, especially to optimize niches for butyrogenic bacteria (Makki et al., 2018; Haak et al., 2019; Maltz et al., 2019). SCFAs help maintain beneficial bacterial populations to support microbial diversity and regulate mucosal barrier integrity, mucosal inflammation, which in turn affects various metabolic functions and nutrient absorption (Mann et al., 2024).

Butyrate once produced, undergoes further metabolism to form glutamate, glutamine, and acetoacetate. Notably, acetoacetate serves as primary energy source for colonocytes supporting their proliferation and differentiation. Butyrate also directly induced mucin expression in polarized goblet cell lines to form a protective mucus layer, acting as a physical barrier shielding epithelial cells from harmful microorganisms and preventing direct contact with the gut contents including ethanol and pro-inflammatory molecules (Martin-Gallausiaux et al., 2021). In addition, SCFAs enhancing epithelial cells tight junctions, which are protein complexes that seals the gaps between cells, by upregulating the expression of protein such as Occludin, Claudins, and Zonula (Zheng et al., 2017).

Microbial SCFAs production is essential to maintain a colonic anaerobic environment (Pral et al., 2021). For instance, butyrate assists in regulating the anaerobic milieu within the colon by activating beta-oxidation in the mitochondria, accounting for over 70% of the oxygen in isolated colonocytes. It achieves this by activating the nuclear receptor peroxisome proliferator-activated receptor gamma (PPARγ) in colonic cells. This activation limits the diffusion of oxygen from colonocytes to the luminal surface, thereby preserving the anaerobic conditions essential for obligate anaerobic organisms (Litvak et al., 2018).

Abundance of literature has extensively documented the supplementation of SCFAs, particularly butyrate, in the treatment of NAFLD and has shown promising results from nutrient metabolism perspectives. Supplementing butyrate for 6 weeks in NAFLD mice model has been shown to alleviate dysbiosis by promoting the abundance of promising bacteria, including *Akkermansia, Roseburia, Coprococcus, Corprobacillus, Delftia, Corynebacterium, Sutterella, Bacteroides, Clostridium*, and *Coriobacteriaceae* populations, while concomitantly reducing the abundance of *Bilophila* and *Rikenellaceae*. Butyrate also has the ability to prevents liver inflammation and injury, as indicated by decreased pro-inflammatory cytokine genes and activation of Kupffer cells in the liver. Furthermore, the gene expression of peroxisome proliferator activated-receptor (PPAR-γ) was upregulated after supplementation, which promotes fatty acid uptake and increases insulin sensitivity (Ye et al., 2018). SCFAs also known to have downstream effects on the endocrine function influencing the secretion of satiety signals hormones such as GLP-1 and PPY, further affecting insulin resistance and lipid metabolism disorder which accompany with NAFLD most of the time (Canfora et al., 2019). Chronic oral butyrate administration prevented diet-induced obesity, hyperinsulinemia, hypertriglyceridemia and hepatic steatosis via expressing neuropeptide Y in the hypothalamus to attribute a reduction in food intake (Li et al., 2018).

Short chain fatty acids has been demonstrated to modulate the balance between fatty acid synthesis, fatty acid oxidation, and lipolysis in the body. SCFAs can modulate various signaling pathways and gene expression, including activation of AMPK, an essential energy-sensing enzyme in the liver. The activation of AMPK has been shown to inhibit the activity of acetyl-CoA carboxylase and reduce the production of malonyl-CoA, which is a precursor to fatty acid synthesis (Canfora et al., 2019). From animal study, nanoparticle-delivered acetate supplementation decreased lipid accumulation, increased mitochondrial efficiency, and inhibited lipolysis. Additionally, acetate supplementation induced “browning” in white adipose tissue which led to a reduction in body adiposity (Sahuri-Arisoylu et al., 2016). In line with these findings, colonic infusions of SCFAs mixture were found to increase fasting fat oxidation and energy expenditure in humans (Canfora et al., 2017). Besides, SCFAs also act as ligands for G-protein coupled receptors (GPCRs), including GPR41 and GPR43 which are expressed in multiple tissues. This interaction has been shown to induce changes in hepatic gene expression that promote fatty acid oxidation and reduce lipogenesis (Neves et al., 2015).

### Bile acids (BAs)

Bile acids are steroid molecules synthesized from cholesterol in the liver forming a critical connection with the gut through multiple mechanisms. Their primary functions extend beyond simple fat digestion and absorption to include metabolic regulation and antimicrobial activity through nuclear receptor signaling (such as FXR and TGR5) (Parséus et al., 2017). In the liver, unconjugated BAs can be processed by conjugating with either taurine or glycine under the action of Bile acyl-CoA synthetase (BACS) and bile acid-CoA:amino acid N-acyltransferase (BAAT) to form conjugated BAs, such as taurocholic acid (TCA) and glycocholic acid (GCA) (Liu et al., 2018). The crosstalk between gut and BAs centers on enterohepatic circulation, where all primary BAs produced by the liver undergo microbial transformation in the gut through processes of deconjugation via bile salt hydrolase (BSH), 7-α-dihydroxylation, oxidation or epimerization, resulting in secondary BAs such as deoxycholic acid (DCA), lithocholic acid (LCA) and ursodeoxycholic acid (UDCA) (Portincasa et al., 2020). Approximately 95% of the BAs in bile acid pool are reabsorbed at the terminal ileum and subsequently recycled back to the liver, establishing a continuous cycle. The amount of BAs that is lost via feces is then replaced by daily BA synthesis. This interaction has profound effects on gut barrier integrity, microbiota composition, and nutrients metabolic function, thus connecting to the progression of NAFLD (Chiang and Ferrell, 2019).

Studies have underscore the importance of the gut microbiome in influencing bile acid signaling and found that primary and secondary BAs were increased in both fecal and serum of patients with NAFLD when compared to healthy controls (Mouzaki et al., 2016). Furthermore, as NAFLD progresses to NASH, ratios of primary to secondary BAs and of conjugated to unconjugated BAs were also increase (Puri et al., 2018). Among conjugated BAs, the GCA to TCA ratio exhibited progressive alterations as it was significantly elevated in NAFL and further increased in NASH but declined sharply upon transition to fibrosis, suggesting altering BSH activity during NAFLD (Chen et al., 2022). Interestingly, plasma taurine levels are decreased in liver disease. Taurine supplementation has shown to reduce pro-inflammatory interleukin expression as well as to maintain the homeostasis of fat metabolism by repressing sterol regulatory element binding proteins-1c (SREBP-1c), thereby alleviating hepatic damage in experimental animal models (Song et al., 2023; Zhu F.-L. et al., 2024). Concurrently, the composition of the gut microbiome was altered in patients with NAFLD, with an 7.2-fold increase increase in bacteria that metabolize taurine and glycine, primarily *Escherichia* and *Bilophila* (Jiao et al., 2018; Puri et al., 2018; Zeng et al., 2024). These bacteria deconjugate TCA via BSH to facilitate overproduction of secondary bile acid DCA, a potent farnesoid X receptor (FXR) antagonist, directly suppressing FXR-driven pathways that regulate hepatic lipid and glucose metabolism.

Bile acids has been recently recognized as an endocrine signaling molecules to activate several nuclear receptors located in the gastrointestinal tract including FXR and G-protein-coupled bile acid receptor -1 (TGR5) to modulate epithelial cell proliferation, intestinal barrier integrity and nutrient metabolism (Adorini and Trauner, 2023). The bidirectional relationship between insulin resistance and NAFLD has been well studied as insulin resistance play a crucial role in the initial pathogenesis of NAFLD and in the progression to NASH (Khan et al., 2019). By activating FXR, increased insulin sensitivity and reduced serum markers of liver inflammation and fibrosis were observed in patients with T2DM and NASH (Mudaliar et al., 2013). Moreover, treatment of fexaramine, a selective FXR agonist, has been shown to alter the gut microbiota, increasing the abundance anaerobic *Acetatfactor* and *Bacteroides*. These two bacteria that have high 7α- and 7-β-HSDH enzymatic activities, enabling them to convert CDCA to LCA. The metabolic benefits occur because LCA are potent activators of TGR5 receptors in the colon and stimulate secretion of GLP-1, which is an incretin that stimulate insulin secretion from pancreatic beta-cells in response to postprandial glucose thereby suggesting improved insulin sensitivity in obese diabetic mice (Kaur et al., 2015; Pathak et al., 2018).

Given the frequent association of NAFLD with obesity, bariatric surgery, as an effective therapy for obesity, has been suggested to substantially change the concentrations of circulating BAs (Chiang, 2015). Chaudhari et al. (2021) demonstrate that LCA, a microbial metabolites secondary bile acid, is increased in murine portal veins following bariatric surgery, which eventually lead to increased GLP-1 secretion. Conversely, LCA levels in the cecum of post-surgery mice exhibited a decrease in stool samples along with the increasing in the abundance of the phyla Bacteroidetes and Proteobacteria (Chaudhari et al., 2021), and a reduction in the abundance of *Clostridia* in the gut (Damms-Machado et al., 2015; McGavigan et al., 2017). The reduction in levels of the LCA in the colon, in surprising contrast to the increase observed in the portal vein suggesting increased reabsorption of BAs. Consistent with increased levels of FGF19 and fasting plasma levels of 6α-hydroxylated BAs, which are TGR5 agonists, in post-surgery human patients, together support that bariatric surgery alters bile acid profiles to improve glucose metabolism (Wahlström et al., 2024).

An emerging area of interest is the interaction between SCFAs and BAs in the gut-liver axis. SCFAs may affect bile acid synthesis and metabolism, influencing liver function and the metabolic outcomes of NAFLD (Zhao et al., 2017; Visekruna and Luu, 2021). The interplay between these metabolites may offer synergistic protective effect against liver conditions by promoting liver metabolism and maintaining bile acid homeostasis.

### Trimethylamine N-oxide (TMAO)

While SCFAs and BAs dominate gut-liver communication, emerging evidence highlights the role of TMAO in exacerbating NALFD progression through multiple aspects.

Trimethylamine N-oxide, a metabolite produced by gut bacteria from dietary components like choline and L-carnitine, has emerged as a significant player in the development and progression of NAFLD. Gut bacteria including Firmicutes and Proteobacteria metabolize these nutrients commonly found in animal-based foods via TMA lyase to produce trimethylamine (TMA), which is then converted to TMAO by flavin monooxygenase in liver. TMA and TMAO levels have been associated with elevated *Firmicute/Bacteroidetes* ratio since *Bacteroidetes* has limited ability to produce TMA. Certain intestinal archaea such as *Methanomassiliicoccales* has the ability to reduce circulating levels of TMAO to methane (Fadhlaoui et al., 2020). The change of gut microbiota composition indicating TMAO synthesis might be attributed to selected bacterial species (Shang et al., 2016). Elevated peripheral blood TMAO levels are positively correlated with major adverse cardiovascular and renal events. Recently, its relation to metabolic condition such as adipose tissue inflammation, T2DM and NAFLD has been identified (León-Mimila et al., 2021; Andrikopoulos et al., 2023; Wang M. et al., 2023).

The production of TMA/TMAO depends on multiple factors beyond gut microbiota, including host genetics and dietary consumption (Meyer, 2020). Since choline serves as a precursor of gut-microbiota-generated TMA, choline rich food play a important source of variability in serum TMAO levels. Western diets which typically contains a large amount of choline rich foods and are known not only to increase blood but also urine TMAO levels (Romano et al., 2015). In contrast, vegetarian and omnivorous diets were associated with lesser ability to produce TMA, along with the gut microbiota composition alteration especially in *Gammaproteobacteria* and *Erysipelotrichi*, reinforcing dietary modulation would have the possible potential to reduce the risk associated with high TMAO levels by limiting liver fat deposition during choline depletion (Lecomte et al., 2015; Tomova et al., 2019).

Elevated TMAO levels can disrupt normal liver function through multiple mechanisms: it interferes with bile acid metabolism and cholesterol homeostasis, impairs the liver’s ability to oxidize fatty acids, and promotes inflammatory responses within liver tissue. *In vivo* and animal studies experiment reveal that TMAO suppresses bile acid production via inhibiting CYP7A1 activity, the key rate-limiting enzyme that oxidize cholesterol to bile acid thus reduce total bile acid pool size (Koeth et al., 2013). In turn, as mentioned earlier, bile acid has the ability to shaping the gut microbiota composition thus influencing intestinal environment that may affect bacterial TMA production. By administering exogenous TMAO *in vitro* and animal models, lipid deposition in HepG2 fatty liver cells was promoted suggesting the relationship between TMAO levels and NAFLD initiation. Elevated TMAO level in mice model further damaged intestinal barrier as evidenced by decreased expression levels of tight junction proteins zona occludens-1 (ZO-1) and Occludin, leading to bacterial infiltration from the gut into liver and lipid accumulation. Besides, higher levels of downstream inflammatory factors, such as TNF-α, IL-1β, and IL-6, and elevated serum lipopolysaccharides (LPS) levels indicating TMAO exacerbates intestinal barrier damage in NAFLD rats, plays a negative regulatory role in disease progression (Nian et al., 2024).

Epigenetic refers to changes in gene expression that do not involve alterations to the underlying DNA sequence. These change can be influenced by environmental factors, lifestyles, and diseases, and provides a new perspective on the pathogenesis of NAFLD (Wu Y.-L. et al., 2023). In NAFLD, abnormal DNA methylation patterns may affect genes involved in lipid metabolism(Chen et al., 2020), inflammation (Lai et al., 2020), and insulin resistance (Baumeier et al., 2017a). For example, mice fed a high fat diet for 6 weeks exhibited reduced methylation of four CpG dinucleotides sites and elevated dipeptidyl peptidase 4 (DPP4) expression. The resulting increase in hepatic DPP4 worsen NAFLD progression by disrupting insulin signaling through autocrine and paracrine mechanisms while decreasing GLP-1 levels (Baumeier et al., 2017b). During the methylation processes, choline serves as a precursor to S-adenosylmethionine (SAM) the primary methyl donor. However, when TMA-producing gut microbiota compete with the host for choline, its bioavailability is significantly reduced to maintain proper methylation processes. This competition ultimately lead to host genome hypomethylation and NAFLD initiation and progression (Romano et al., 2017). In addition, acetate can also influence histone acetylation through Acetyl-CoA Synthetase Short Chain Family Member 2 (ACSS2), a nucleo-cytosolic enzyme involved in fat deposition and disrupted cellular signaling in the context of NAFLD, leading to hepatic steatosis or inflammation (El-Kurjieh et al., 2025).

This insight suggests the possibility of using individualized dietary and nutrient interventions to modulate both gut microbiota composition and specific metabolic pathway, including TMAO and acetate synthesis in order to offer promising therapeutic approaches for treating NAFLD and its complications.

## The role of probiotic supplementation

Due to the close relationship between the liver and gastrointestinal tract, it is not surprising that restoring a healthy gut microbiome composition and abundance through targeted interventions such as probiotics, prebiotics, phytochemicals and dietary changes, has the potential to combat the overgrowth of harmful bacteria, bolster the intestinal mucosal barrier, and eventually reduce fat accumulation, and mitigate liver damage. Probiotics defined as “live microorganisms that, when administered in adequate amounts, confer a health benefit,” have been shown to play a significant role in the pathogenesis and progression of NAFLD, as mentioned in this review. Accumulating studies have demonstrated the feasibility of certain probiotic supplementation including *Lactobacillus, Bifidobacterium, Polycoccus and Streptococcus* against NAFLD and NASH in human and in mouse models (Yoo and Kim, 2016).

As a widely used probiotic in food industry, *Lactobacillus acidophilus* has been drawn much attention *L. acidophilus* NCFM has the potential to improve insulin sensitivity and inflammatory response (Adams et al., 2005). In a separate study, *L. acidophilus SNZ 86* was found to ameliorate helps NAFLD in rats induced by western diet by activating AMPK/SIRT-1 signaling pathway, which increase fatty acid oxidation, reduces lipogenesis in the liver, and improve insulin sensitivity (Pant et al., 2023). *L. acidophilus KLDS1.0901* administration followed by high-fat diet effectively restored the increased concentrations of cytokines including IL-6, IL-1β, and TNF-α, as well as lowered lipid content in levels of total cholesterol, triglyceride (TG), and low-density lipoprotein cholesterol (Wang Y. et al., 2023). A different species of L. plantarum NA136 also has elucidated the therapeutic potential in mitigating NAFLD via targeting the AMPK/Nrf2 pathway and suppressing the SREBP-1c/FAS signaling pathway in order to inhibit adipogenesis from preadipocytes (Zhao et al., 2020).

Oxidative stress refers to an imbalance between the production of reactive oxygen species and the body’s ability to neutralize them with antioxidants, implicate a key factor in early pathogenesis of NAFLD (Parthasarathy et al., 2020). *In vivo*, *L. plantarum* ATG-K2 and *L. plantarum* ATG-K6 enhanced antioxidant activity by regulating the expression of antioxidant enzymes like superoxide dismutase (SOD), glutathione peroxidase (GPx) and catalase (CAT) via the Nrf2/Keap1 signaling pathway, a key system for regulating the expression of antioxidant and cell-protective genes (Park et al., 2020). Upon activation of Nrf2, its ability to enter the cell nucleus and bind to the antioxidant response element (ARE), thereby promoting the expression of antioxidant enzymes, thereby reducing oxidative stress-induced damage to the liver (Park et al., 2021).

During the progression from NAFLD to NASH, the dysregulation of different innate and adaptive immune cells plays an integral role, of which natural killer (NK) cells are an example. Supplementation of *Bifidobacterium uniformis* and *Bifidobacterium bifidum* for 8 weeks significantly upregulate the expression of the NKG2D receptor located on the surface of NK cells and to promote the secretion of cytotoxic enzymes by NK cells, such as granzyme B and perforin to induce apoptosis of target cells directly. NKG2D is an important activating receptor of NK cells, and its ligand recognition can enhance the cytotoxicity and the killing ability of NK cells (Xu et al., 2024). In an animal model, intervention with *Bifidobacterium longum* has been linked to increased serotonin (5-HT) levels in both serum and feces, attributable to elevated expressions of cyclin D1 and Ki67, pivotal regulators of the transition from the G1 phase to the S phase of the cell cycle. Elevated expression promotes liver cellular proliferation and reduce the formation of scar tissue. Furthermore, an increase in secondary BAs and butyric acid levels after intervention were observed which is in line with the diminishes the expression of proinflammatory factors TNF-a, IL-1b, f4/80 (Yu et al., 2024). A summary of current probiotic strains associated with treatment of NAFLD is shown in Table 2.

**TABLE 2:** Summary of probiotic strains and mechanisms in the treatment of NAFLD.

Level of genus	Probiotic strains	Announced strain name	Dosage	Intervention	Mechanism	References
*Lactobacillus*	*Lactobacillus rhamnosus GG*	*L. rhamnosus GG (ATCC 53103), L. acidophilus (ATCC 4356), L. gasseri (ATCC 33323)*	1 × 10^9^ CFU/day	9 weeks	Regulate lipid metabolism:mRNA expression of lipid synthesis genes, Dgat1 and Dgat2;	Jang et al., 2019
		*Lactobacillus rhamnosus GG*	1 × 10^9^ CFU/day	11 days	Bile acid metabolism: inhibition of liver BAs synthesis through intestinal FXR-FGF15 signaling increasing BSH containing gut bacteria to excrete BAs	Liu et al., 2020
		*Lactobacillus rhamnosus GG (ATCC 53103)*	1 × 10^9^ CFU/day	10 days	Maintain intestinal barrier:increased the number of goblet cells; upregulated expression of 5-HT4R and MUC2	Gu et al., 2022
		*Lactobacillus rhamnosus GG*	1 × 10^9^ CFU/day	6 weeks	Anti-inflammation: restore the activity of antioxidant enzymes; inhibit TLR4/NF-κB pathway, decrease TNF-α and IL-6	Arellano-García et al., 2023
	*Lactobacillus plantarum*	*Lactiplantibacillus plantarum AR113*	1 × 10^9^ CFU/ml	2 weeks	Accelerate liver cell regeneration: increased tumor necrosis factor-α (TNF-α), hepatocyte growth factor (HGF), and transforming growth factor-β (TGF-β) expression	Xie et al., 2021
		*Lactobacillus plantarum MA2*	1 × 10^9^ CFU/g.bw	56 days	Repairs intestinal barrier: upregulates Ocln, Muc2, and ZO-1 expression; reduce LPS circulation	Wang Y. et al., 2021
		*Lactobacillus plantarum LP104*	1 × 10^9^ CFU/ml × 5 ml	8 weeks	Lipid metabolism: inhibits lipid formation and promotes lipolysis through SREBP-1/PPARα signaling pathway	Teng et al., 2022
		*L. plantarum ATG-K2* *L. plantarum ATG-K6*	K2: 5 × 10^8^ CFU/day K6: 5 × 10^8^ CFU/day	8 weeks	Regulating Microbiota Composition: increased the ratio of Firmicutes to Bacteroidetes, decreased the abundance of Proteobacteria	Park et al., 2020
		*L. plantarum NA136*	1 × 10^9^ CFU/day	16 weeks	Regulating Microbiota Composition: promoting growth of *Allobaculum*, *Lactobacillus*, and *Bifidobacterium*; Anti-inflammation: inhibited serum LPS, TNF-α, IL-6, and IL-1β levels via decreased NF-κB P65 protein expression	Zhao et al., 2020
	*Lactobacillus casei Shirota*	*Lactobacillus casei Shirota*	3 × 10^10^ CFU/day	4 weeks	Regualte SCFAs production: increase total SCFA and propionic acid contents	Joseph et al., 2019
		*Lactobacillus casei Shirota*	6.5 × 10^9^ CFU/bottle × 3/day	6 months	Anti-inflammation: decrease in IL-1β, MCP-1, IL-17A and MIP-1β	Macnaughtan et al., 2020
*Bifidobacterium*	*Bifidobacterium longum*	*Bifidobacterium lon gum and Fructo-oligosaccharides*	*Bifidobacterium longum* and Fos 2.5 g, vitamin B1 (1.4 mg), vitamin B2 (1.6 mg), vitamin B6 (2.0 mg), and vitamin B12 (1.0 mg)	24 weeks	Suppression of endotoxin:reduces the production and absorption of intestinal toxins	Malaguarnera et al., 2012
		*Bifidobacterium longum, Lactobacillus acidophilus, Enterococcus faecalis*	8 × 10^7^ CFU/day of each bacterium	8 days	Modulate microbiome metabolites: increase secondary BAs and butyric acid levels; Anti-inflammation: downregulated expressions of TNF-α, IL-1β, and f4/80; Accelerate liver cell regeneration: upregulated expressions of cyclin D1 and Ki67; Regulating Microbiota Composition: increased aboundance of *Bifidobacterium* and *Lachnospiraceae*	Yu et al., 2024
	*Bifidobacterium breve*	*Bifidobacterium breve BBr60*	1 × 10^10^ CFU/day	12 weeks	Regulating Microbiota Composition: increase in α diversity; increase the abundance of *Escherichia-Shigella, Dialister, Phascolarctobacterium, Klebsiella, Bacteroides*, and *Veillonella*; Improving clinical indicator: reduction in body weight, body fat percentage, waist-to-hip ratio, fasting blood glucose, LDL-C, ALT, AST; increase in HDL-C; Nutrient metabolism: regulated amino acids metabolism and citrate cycle metabolism	Bai et al., 2024
*Saccharomyces*	*Saccharomyces boulardii*	*Saccharomyces cerevisiae var. boulardii*	7 × 10^10^ CFU of *Lactiplantibacillus plantarum* 299v 5 × 10^9^ CFU of *Saccharomyces cerevisiae var. boulardii*	12 weeks	Anti-inflammation: downregulated expressions of CRP, IL-6; Regulate appetite hormones: increase ghrelin level via growth of SCFA-producing microbes	Okuka et al., 2024
*Akkermansia*	*Akkermansia muciniphila*	*A. muciniphila DSM 22959*	OD value of 0.15 was 10^8^ CFU/mL	48 hours	Repairs intestinal barrier: increased MUC2 secretion; upregulates Ocln, Muc2, and ZO-1 expression	Liu et al., 2021
		*Akkermansia muciniphila MucT*	2 × 10^7^ bacterial cells	10–12 hours	Modulate microbiome metabolites: support the growth of *Faecalibacterium prausnitzii* and *Roseburia* spp. To produce SCFAs, succinate and 1,2-propanediol	Ottman et al., 2017
		*A. muciniphila*	1 × 10^9^CFU/day	20 weeks	Immunomodulation: inhibite kynurenine pathway; reduce hepatic macrophages (M1) and γδT17 cell	Han et al., 2023
		*A. muciniphila BAA-835*	1 × 10^8^ to 1 × 10^9^ CFU/ml	10 weeks	Lipid metabolism: suppressing SREBP expression	Kim S. et al., 2020
		*Pasteurized Akkermansia muciniphila*	2 × 10^8^ CFU/day	5 weeks	Improved glucose tolerance: decreases glucose and fructose absorption via downregulate GLUT2, GLUT5 and SGLT1 mRNA expression;	Ottman et al., 2017
Postbiotics	*Lactobacillus plantarum-derived extracellular vesicle*	*L. plantarum APsulloc*	≥2.7 × 10^9^ CFU/ml	48 hours	Immunomodulation: restores the M1/M2 imbalance	Kim W. et al., 2020
Probiotic mix	*Lactobacillus* and *Bifidobacterium*	*Lactobacillus acidophilus, Lactobacillus casei subsp., Lactobacillus lactis, Bifidobacterium bifidum, Bifidobacterium infantis and Bifidobacterium longum*	6 × 10^10^ CFU/day	6 months	Mucosal immune function: stabilize CD8+ T lymphocyte count	Mohamad Nor et al., 2021

### Emerging therapeutic candidate: *Akkermansia muciniphila*

Since its discovery two decades ago, *Akkermansia muciniphila*, a Gram-negative anaerobic bacterium inhabiting the mucus layer of the gastrointestinal tract has rapidly attracted widespread attention from both the academic and industrial communities due to its potential probiotic beneficial effects in various metabolic diseases, and it is expected to become a star strain of the next-generation therapeutic probiotic. The genus *Akkermansia* begins to colonize in the early stage of human life, and its relative abundance gradually increases during infancy and early childhood (Collado et al., 2007). During the growth process, the intake of high-fiber foods, polyphenol-rich foods and caloric restriction diet pattern can indeed affect the abundance of *A. muciniphila* (von Schwartzenberg et al., 2021). Unlike many gut bacteria, *A. muciniphila* trives in mucin degradation and can produce enzymes that degrade oligosaccharide chains, such as glycosidases, sulfatases, and sialidases, to adapt to the living environment rich in mucin and endogenous glycoproteins in the mucus layer (Liu et al., 2021). At the meantime, the thickness of the mucus layer is related to the abundance of *A. muciniphila* in the gastrointestinal tract.

A substantial body of research has been dedicated to elucidating the mechanisms through which *A. muciniphila* exert its systematic influences in the liver of NAFLD. The emergence of *A. muciniphila* has been demonstrated to modulate gut microbiota, strengthening the intestinal barrier, and regulate immune response that related to NAFLD and its progression to NASH.

*Akkermansia muciniphila* repairs intestinal barrier function by stimulating mucin production, enhancing tight junction integrity, and modulating immune responses. It promotes the activity of goblet cells, leading to increased secretion of mucins (MUC2), which reinforce the protective mucus layer and prevent harmful microbes and toxins from reaching the gut epithelium (Kim et al., 2021; Liu et al., 2021). Additionally, *A. muciniphila* upregulates the expression of tight junction proteins Ocln, Muc2, and ZO-1, strengthening epithelial cell connections and reducing intestinal permeability.

Furthermore, during the process of mucin degradation, *A. muciniphila* releases oligosaccharides and simple sugars that can serve as substrates for other gut bacteria, including SCFA producers *Faecalibacterium prausnitzii* and *Roseburia spp.* for further fermentation resulting in the production of SCFAs, succinate and 1,2-propanediol (Ottman et al., 2017; Pichler et al., 2020). In exchange, *A. muciniphila* receives a vitamin B12 analog as an essential cofactor in DNA synthesis from other bacteria indicating a bidirectional cross-feeding between different microbial species (Ioannou et al., 2024). These mucin metabolites contribute to host’s metabolic health, with acetate and propionate playing key role in modulation of gut hormones like peptide YY and GLP-1, as well as systemic hormones like insulin and glucagon, influencing appetite control and gastric emptying (Hernández et al., 2019; Bridgeman et al., 2020). Besides stimulating SCFAs production, emerging researches have shown that the gut microbiota supports intestinal barrier protection by modulating BAs and tryptophan derivatives (Michaudel and Sokol, 2020; Wu W. et al., 2023). Administration of *A. muciniphila* reversed the level of tryptophan metabolites such as 3-hydroxykynurenine and 5-hydroxykynurenine, as well as upregulated the gene expression of enzymes in kynurenine pathway which is a primary route of tryptophan degradation in the liver (Han et al., 2023). The kynurenine pathway have been shown to interact with aryl hydrocarbon receptors (AhR), which regulate the function of T cells and macrophages as well as differentiation of Th17cells that contribute to liver inflammation and fibrosis in NASH (Teunis et al., 2022).

In both animal and human trials, high-fat diet and metabolic disease condition are significantly related to the relative gut abundance reduction of *A. muciniphila* (Rao et al., 2021; Yoon et al., 2021; Zhang et al., 2021; Li T. et al., 2023). *A. muciniphila* supplementation was found to positively impact lipid metabolism and prevent NAFLD in mice through regulation of genes involved in lipogenesis and the expression of pro-inflammatory cytokines in liver tissue (Han et al., 2023). An additional study revealed that *A. muciniphila* specifically regulates the transcription of sterol regulatory element-binding protein (SREBP), a family of transcription factors that regulate lipid homoeostasis, to protect liver fat accumulation (Kim S. et al., 2020). *A. muciniphila* has also been shown to modulate the immune system, reducing hepatic proinflammatory macrophages (M1) and γδT and γδT17 cells in high-fat diet-induced NASH mice. The shift from M1 macrophages to M2 macrophages in the liver were partly regulated by *A. muciniphila* administration to promoting its anti-inflammatory effect (Han et al., 2023). Futhermore, breast milk-isolated *A. muciniphila* were shown to prevent NASH from progressing to hepatocellular carcinoma through modulation of immune system, specifically CXCR6+ natural killer T (NKT) cells, thereby enhancing its ability to counteract inflammation-driven tumorigenesis (Li T. et al., 2023).

Interestingly, even pasteurization of *A. muciniphila* also promisingly exert similar positive effects in mice (Depommier et al., 2020; Wu et al., 2022; Wang Y. et al., 2024). The benefits mostly link to the outer cell membrane protein Amuc-1100 of *A. muciniphila.* Amuc-1100 has been observed to interact with immune cells, particularly through Toll-like receptors (TLRs) present on intestinal and liver immune cells, leading to a reduction in systemic inflammation. This has been shown to improve fatty acid oxidation and insulin sensitivity, both of which are critical for managing NAFLD (Cani et al., 2022). Notably, comparing to live form, pasteurized form is more effective in upregulating tight junction proteins expression and in inducing higher levels of SCFAs in the ileum (Grajeda-Iglesias et al., 2021). Meanwhile, both alive and pasteurized *A. muciniphila* promote the growth of beneficial bacteria while reducing the abundance of harmful microbes. This shift in microbial composition contributes to a healthier gut ecosystem, reducing the production of metabolites that could exacerbate inflammation and liver dysfunction (Ashrafian et al., 2021).

Pasteurized *A. muciniphila* offers significant advantages in the development of novel probiotic products with high commercial value (Plovier et al., 2017). As the bacteria are inactivated, pasteurized *A. muciniphila* cannot reproduce, eliminating the risk of infection and making them safer for individuals with weakened immune systems (Turck et al., 2021). Additionally, pasteurized *A. muciniphila* is also more stable during storage and transportation, reducing the need for strict environmental controls and extending its shelf life (Abbasi et al., 2024). Despite these advantages, unlike live bacteria, it lacks the ability to colonize the gut, meaning its effects may be short-lived and require higher doses or more frequent administration to achieve results comparable to live bacteria (Wang B. et al., 2023; Xie et al., 2023; Liu et al., 2024). It highlights the need to carefully consider the patient’s needs when choosing between live and pasteurized supplementation.

## Therapeutic potential of other compounds for NAFLD

In addition to the above-mentioned probiotics, prebiotics and certain phytochemicals have also played a positive role in therapeutic potential of NAFLD. Prebiotics are selectively fermented, non-digestible dietary compounds such as inulin, oligosaccharides, have been shown to positively influence the gut microbiota, which is closely linked to NAFLD pathogenesis. The supplementation of oligofructose for 36 weeks has shown statistically significant reduction in hepatic steatosis and NASH score, as well as to improve gut microbiome diversity and to modulate gut microbiota profile (Bomhof et al., 2019; Carpi et al., 2022). Prebiotics exert their beneficial effects by enhancing the growth of beneficial gut bacteria to favor SCFAs production, improving gut barrier function, reducing endotoxemia and modulating inflammatory responses.

Recent research has revealed that certain plant-derived compounds can have a significant impact on NAFLD by modulating the gut microbiota. Nuciferine, a bioactive compound found in lotus leaves, has been shown to enhance gut barrier integrity by upregulating tight junction protein expression and suppressing inflammation via TLR4/MyD88/NF-κB pathway (Zhu X. et al. 2024). Similarly, theabrownin, derived from Pu-erh tea, acts as an intestinal farnesoid X receptor (FXR) antagonist to mitigate NAFLD by inhibiting the intestinal FXR-ceramide axis. The reduction of ceramide levels in the intestine and liver further downregulates ceramide synthase expression, thereby decreasing hepatic lipid accumulation and improving hepatic steatosis in animal models (Wang J. et al., 2024). While these two plant-derived compounds show potential in improving NAFLD by beneficially modulating the gut microbiota. However, excessive consumption of longan fruit, which is rich in free sugars, has been found to disrupt gut homeostasis. It reduces the Bacteroidetes/Firmicutes ratio, increases potentially pathogenic bacteria, decreases beneficial bacteria, and reduces SCFA production, thereby promoting NAFLD development (Wu X. et al., 2023). This indicates that the impact of plant-derived compounds on NAFLD is dual-edged, with effects varying depending on the type and dose of the compound.

Tauroursodeoxycholic Acid (TUDCA), a synthetic bile acid derived from the conjugation of UDCA with taurine, has been widely recognized for its therapeutic potential in cholestatic liver diseases (Torres et al., 2019). Beyond its conventional uses, TUDCA intervention is positively associated with the abundance of beneficial bacteria such as *Allobaculum* and *Bifidobacterium* in NAFLD animal models. It also influences bile acid metabolism by upregulating key enzymes cholesterol 7α-hydroxylase (CYP7A1) and sodium taurocholate cotransporting polypeptide (NTCP). This results in increased hepatic bile acid levels, which facilitate cholesterol conversion to BAs and promote their enterohepatic circulation, thereby alleviating hepatic lipid accumulation (Wang H. et al., 2024). Medium to high dose of TUDCA (50 and 100 mg/kg/day) have shown to enhance hepatocyte proliferation by upregulating GATA3 activity, and to alleviates liver fibrosis by attenuating hepatic stellate cell activation in animal study (Bai et al., 2025). These findings underscore the significant therapeutic potential of synthetic BAs compounds in NAFLD treatment.

## Bridging the gaps: current limitations

Despite the promising findings, there are significant limitations in current research that need to be addressed. First, there is significant heterogeneity among human studies in terms of design protocols, diagnostic criteria, patient population characteristics, and methods of flora analysis, making cross-study comparisons difficult. Second, although microbial-derived metabolites (e.g., SCFAs, BAs, TMAO) have been demonstrated to be associated with NAFLD pathogenesis, their clinical translational application has not been clarified and causality remains unestablished. Human intervention trials with isotope-labeled metabolites or fecal microbiota transplantation (FMT) are needed to validate mechanisms. In addition, current microbiota studies primarily rely on 16S rRNA sequencing, which lacks resolution at the species level and functional insights. While metagenomics resolves these limitations, its high cost and computational demands hinder widespread clinical application. More importantly, comprehensive databases integrating microbiome and multi-omics data (e.g., metabolomics, lipidomics) as well as disease phenotypes have not yet been developed. Future studies should integrate multi-omics approaches including metagenomics or metatranscriptomics to link specific flora to NAFLD progression. This review has not addressed translational applications in clinical practice or causal relationships supported by existing databases. Given the existing methodological limitations, screening of diagnostic markers through computational science and integrated multi-omics approaches represents a critical direction of exploration in the future.

## Conclusion

Non-alcoholic fatty liver disease is a multifactorial metabolic disorder with significant public health implications, potentially progressing to NASH, fibrosis, and even hepatocellular carcinoma. The progression of NAFLD is driven by a complex interplay between genetic, metabolic, and environmental factors drives its progression, with the gut-liver axis emerging as a critical pathway in its pathogenesis. Accumulating evidence highlights the pivotal role of the gut microbiota in modulating hepatic lipid deposition, inflammation, and insulin resistance through microbiota-derived metabolites, including SCFAs, BAs, and TMAO offering new insights into the progression of the disease. Emerging therapeutic strategies targeting the gut microbiota offer promising avenues for NAFLD management. Probiotics, such as *Lactobacillus, Bifidobacterium*, and *Akkermansia muciniphila*, have shown significant benefits in restoring microbial balance, enhancing gut barrier function, modulating nutrient metabolism, and reducing inflammation. The integration of probiotics, prebiotics, plant-derived compound, and TUDCA leverages the complementary mechanisms to address the multifactorial nature of NAFLD and to position them as next-generation probiotics for NAFLD treatment. Future research could focus on optimizing the combinations and dosages of these therapies, validating their efficacy in large-scale clinical trials, and exploring personalized treatment regimens based on individual gut microbiota profiles.

In summary, the targeting of the gut microbiota and its metabolites presents a promising strategy for the prevention and treatment of NAFLD. However, challenges persist. The heterogeneity of NAFLD and the complex interactions between the gut microbiota and host metabolism underscore the necessity for personalized therapeutic approaches. Additionally, the long-term efficacy and safety of probiotic interventions, particularly in diverse patient populations, require rigorous evaluation. Consequently, there is an imperative for further research to develop precision-based therapeutic approaches, and exploring the potential synergistic effects of combining probiotics, prebiotics, plant-derived compounds and BAs with existing pharmacological treatments in managing this multifaceted disease.
